# Bovine Meat and Milk Factors (BMMFs): Their Proposed Role in Common Human Cancers and Type 2 Diabetes Mellitus

**DOI:** 10.3390/cancers13215407

**Published:** 2021-10-28

**Authors:** Ethel-Michele de Villiers, Harald zur Hausen

**Affiliations:** Deutsches Krebsforschungszentrum (DKFZ), Im Neuenheimer Feld 280, 69120 Heidelberg, Germany

**Keywords:** indirect carcinogenesis, bovine meat and milk factors (BMMF), chronic zoonosis

## Abstract

**Simple Summary:**

This manuscript emphasizes the mechanistic differences of infectious agents contributing to human cancers either by “direct” or “indirect” interactions. The epidemiology of cancers linked to direct carcinogens differs (e.g., response to immunosuppression) from those cancers linked with indirect infectious interactions. We discuss their role in colon, breast, and prostate cancers and type II diabetes mellitus. A brief discussion covers the potential role of BMMF (bovine meat and milk factor) infections in acute myeloid leukemia.

**Abstract:**

Exemplified by infections with bovine meat and milk factors (BMMFs), this manuscript emphasizes the different mechanistic aspects of infectious agents contributing to human cancers by “direct” or “indirect” interactions. The epidemiology of cancers linked to direct carcinogens (e.g., response to immunosuppression) differs from those cancers linked with indirect infectious interactions. Cancers induced by direct infectious carcinogens commonly increase under immunosuppression, whereas the cancer risk by indirect carcinogens is reduced. This influences their responses to preventive and therapeutic interferences. In addition, we discuss their role in colon, breast and prostate cancers and type II diabetes mellitus. A brief discussion covers the potential role of BMMF infections in acute myeloid leukemia.

## 1. Introduction:

Viral, bacterial, or parasitic infections contribute, as causative agents, to a number of human cancers [1]. Most of these reports have noted 14–16% as the global cancer incidence caused by infectious events—many caused by persisting tumor virus infections [2]. Our group calculated slightly higher incidence rates (20–21%) [1]. Basic criteria considered here for definition of a causal role of infections in cancers were:

(1) Persistence of whole genomes or specific genes in certain cancer cells;
(2) Transformed or malignant phenotype of the latter, dependent on the expression and/or function of those genes;
(3) Induction of malignant growth in susceptible animal systems.

**Figure 1:**
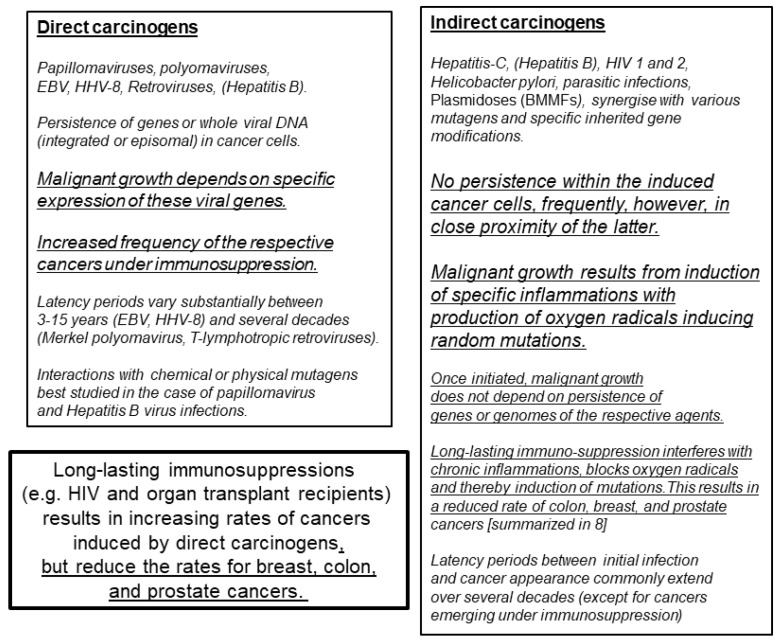
Mechanisms by which infections contribute to human cancer development. BMMFs (bovine meat and milk factors) represent small single-stranded circular DNA, predominantly isolated from sera and dairy products of Eurasian cattle and subsequently identified in periglandular cells of colon, breast and prostate cancers [6]. These infectious agents share characteristics of both bacterial plasmids and known viruses [9], and are related to two DNA isolates from transmissible spongiform encephalopathies reported by Manuelidis [10].

These, among others (Figure 1), are common features of *direct carcinogenesis* and evidence for a link between specific infections and cancer [1,3,4]. A few exceptions, however, did not fit these postulates—for instance, the absence of hepatitis C virus in liver cancer cells of patients at high risk after long-time exposure to this persistent virus. In cases where infections lead to chronic inflammation, cancers eventually arise locally, although the infectious agent itself does not persist in the respective cancer cells. Helicobacter pylori infections may lead to chronic inflammation, ulcers and eventually gastric cancers. Parasitic infections of the vascular system in the bladder and liver may eventually lead to bladder and liver cancer, respectively [1]. The role of chronic inflammations emerges as relatively non-specific or indirect by inducing the production of reactive oxygen molecules, leading to increased mutagenesis at the respective sites [5,6]. Support for this view originated from observations revealing increased cancer risk in long persisting chronic ulcerations, scar tissue and even in poxvirus vaccination scars [7]. It has been difficult to identify such infectious factors, as well as to understand the mechanism in detail of their contribution to different types of cancers.

Presently established differences between direct and indirect modes of carcinogens are defined in Figure 1 (summarized from [1,3,4,8]). Further evaluation is required to analyze whether the role of hepatitis B infection (hepatitis B in both columns) in carcinogenesis is direct and/or indirect.

Recently, several publications reported viral detection after whole-genome DNA sequencing of a large number of different human cancers [11,12,13,14]. These studies aimed at identifying hitherto unknown tumor viruses, in part defined by their close relationship to known oncogenic agents. In summary, taking aspects known for direct carcinogenic involvement into consideration, the majority of results failed to identify additional known or unknown foreign DNA sequences in the tumors investigated. This resulted in concluding that at least the majority of infectious agents contributing as direct carcinogens to human cancers is presently known [15]. The authors supported their argument with observations that cancers with persisting foreign DNA are commonly substantially increased in incidence during immunosuppression following organ transplantation or persistent human immunodeficiency infections. This is in remarkable contrast to other common cancers (e.g., colon, breast and prostate cancers), which do not increase under the same conditions [8]. In contrast to cancers linked to direct carcinogens, immunosuppression was even protective against these latter cancers (Figure 1).

## 2. Bovine Meat and Milk Factors (BMMFs)

Epidemiological studies have pointed to a relationship between colon, breast and prostate cancers and nutritional habits—in particular, to red meat consumption. We evaluated different geographic risks for colon and breast cancers in relation to meat consumption [16]. Surprising results pointed to differences involving consumption of red meat originating from different species of cattle, e.g., Eurasian dairy cattle versus Zebu and Yak breeds [16]. Countries consuming red meat mostly from Eurasian dairy cattle origins revealed high incidences of breast and colon cancers. Mongolia was an outstanding exception [17]. Residents of this country traditionally consume high quantities of red meat (from Yak or Chinese Yellow cattle origin), but incidences of colon and breast cancers are remarkably low. Wide variations in colon cancer incidences have been reported, depending on specific life-style habits [18]. Globocan calculations of 2008 calculate an age-standardized risk in Western populations of <38 per 100,000 inhabitants. In Mongolia the respective data are< 3.2 per 100,000 inhabitants. Thus, the difference in incidence seems to exceed a factor of 10. Less conspicuous observations exist in a few other countries. Recently, however, BMMF 1 and 2 sequences were demonstrated in milk from a different aurochs-derived bovine species: water buffalos (*Bubalus arnee f. bubalis*) [19].

**Figure 2:**
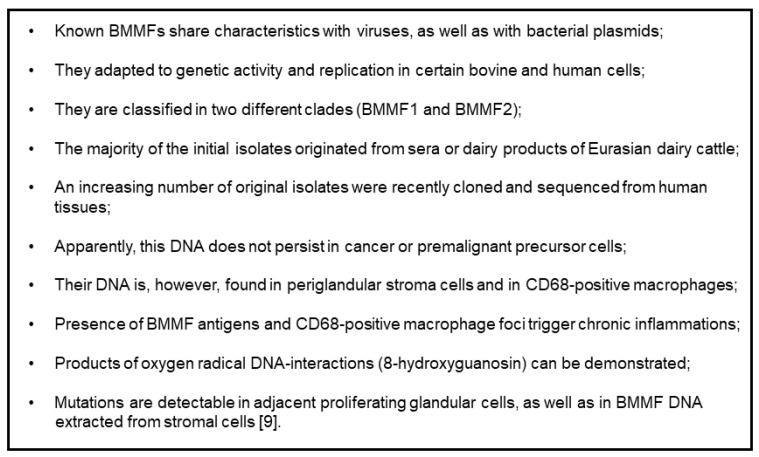
Present knowledge concerning BMMFs (molecular and immunologic evidence).

**Figure 3:**
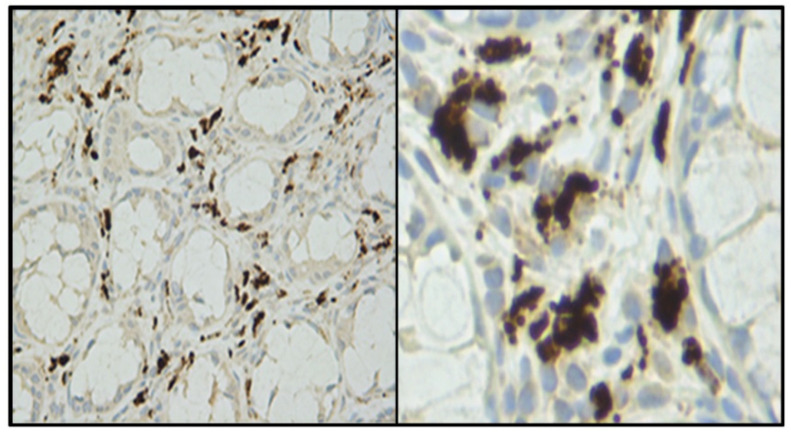
Typical staining pattern of cells expressing the BMMF replication protein, surrounding Lieberkühn crypts of a colon cancer biopsy at 20× and 40× magnification. Photos provided by Timo Bund.

Our hypothesis [7,16], linking high-risk regions for colon and breast cancers and preferential consumption of Eurasian dairy cattle meat and milk products, prompted experimental studies searching for possible nutritional infectious factors as responsible carcinogens. Thus, we tried to isolate and identify potential agents linked to colon and breast cancers from the milk, dairy products, and serum of Eurasian dairy cattle [9,20,21]. Based on our previous studies with human TT viruses, we concentrated on the isolation of small circular single-stranded DNA. A large number of these molecules, grouped in at least two clades, all share characteristics attributed to both bacterial plasmids, as well as viruses (Figure 2) [9]. The development of monoclonal antibodies against the protein derived from the largest open reading frame, identified in silico as a replication gene (Rep), proved to be very helpful in analyzing the tissue localization and the cell types expressing this protein (Figure 3) [22].

**Figure 4:**
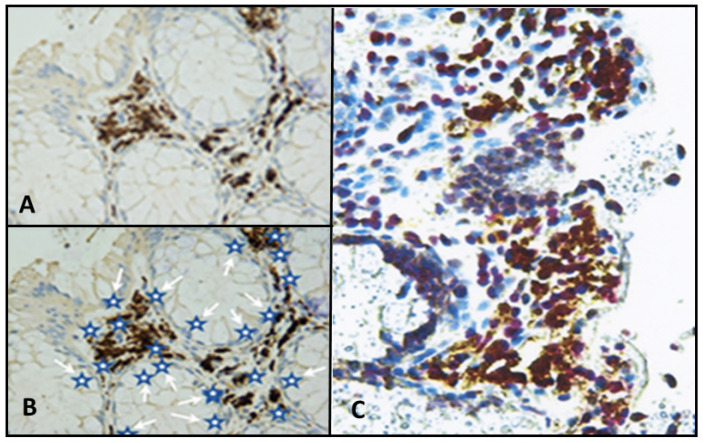
(**A**) Lamina propria cells surrounding Lieberkühn’s crypts of the colon, staining positive with monoclonal anti-Rep antibodies. (**B**) Schematic representation of active sites of inflammation-caused oxygen radical activity indicated by white arrows pointing to blue stars. (**C**) Experimental confirmation staining of Rep-positive lamina propria cells with antibodies directed against 8-hydroxy-guanosin as a marker for oxygen radical activity (yellow staining). Reproduced [6] with permission.

The obtained results (Figure 4) pointed to an indirect mode of BMMF action in carcinogenesis, as previously discussed in detail [6]. In addition, Rep antigen staining occurred in non-glandular tissue of breast and prostate cancer biopsies [23].

In a recent report, Zapatka et al. [14] published a supporting table (without referral in the text) containing contig data of BMMF sequences obtained after sequencing colon, breast, prostate and other cancer biopsies. The authors identified the sequences according to an outdated NCBI databank submission (2014) [14], describing them mainly as “sphinx-related 1.74 or 2.36 contigs”. An updated analysis of these sequences showed almost all of them as corresponding to sections of complete BMMF1 or BMMF2 genomes present in all databanks and as previously published [9]. Although not referencing these latter data, Zapatka et al. [14] confirm our colon and breast cancer studies and even extend our data by including a few additional cancers.

## 3. Potential Consequences of Indirect Carcinogenesis for Strategies in Prevention and Therapy of BMMF-Linked Cancers

In virus-induced direct carcinogenesis, uptake and expression of foreign genes from infectious agents (e.g., cervical cancer, Kaposi’s sarcomas, Burkitt’s lymphomas and others) lead to growth stimulation and increased mutagenic activity [6]. In addition, the continued interference of one or more viral proteins with growth-regulating host cell genes is a requirement for malignant transition and maintenance of the transformed phenotype [1]. These transformed cells are recognized by the immune system of the respective host, leading to inflammatory infiltrates of tumor-associated macrophages (TAM), in addition to T- and B-lymphocytes, which surround islands of a number of solid human cancers. They seem to act as a natural defense mechanism against uncontrollable cell proliferation [24,25]. The majority of these cancers arise at high frequencies under severe immunosuppression [15].

**Figure 5:**
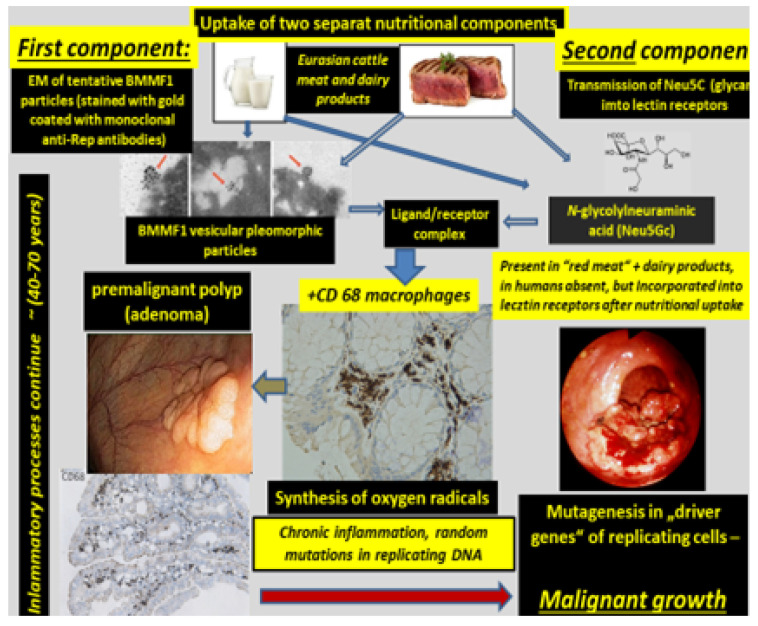
Sequence of events leading to malignant growth in colon. Uptake of two components is required for binding of infectious particles of bovine meat and milk factors (BMMF) to Neu5Gc as a terminal component of lectin receptors. Both are commonly present in Eurasian bovine sera and dairy products. EM—electron microscopy.

**Figure 6:**
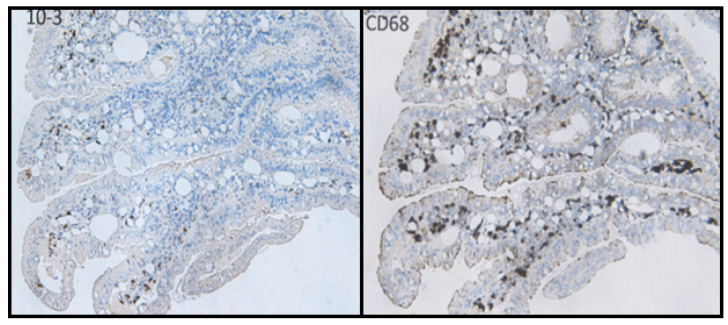
Colon polyps stained with anti-Rep and anti-CD68 monoclonal antibodies. The continuation of inflammation alongside mutagenic activity is evidenced by CD68 presence and Rep expression.

Cancers resulting from an infectious cause and linked to indirect modes of carcinogenesis follow a different pattern. The sequence of events, as evidenced by BMMF infections and cancer of the colon, was, as previously proposed [6], to be the following (Figure 5):

1. Uptake of infectious agents by nutrition through dairy products and/or meat of Eurasian cattle.
2. Infection by these agents and expression of their antigens in lamina propria cells (stromal mesenchymal cells and CD68-positive macrophages).
3. Macrophage-mediated inflammatory response (reactive oxygen production).
4. Random mutagenesis in DNA-replicating Lieberkühn crypt cells adjacent to infected cells, as well as in replicating single-stranded BMMF-DNA.
5. After long latency periods (commonly more than three decades) “driver”-mutations in genes of specific cells are established and enhanced growth of such clones occurs. These clones undergo further mutations in two or more additional steps, leading to the development of premalignant polyps. Final transformation of these polyps into malignant tumors occurs due to continuing mutagenic activity. This follows a pattern outlined previously and summarized by Greaves and Maley [26] in a quote: “Cancers evolve by a reiterative process of clonal expansion, genetic diversification and clonal selection within the adaptive landscapes of tissue ecosystems”.
6. It is evident from the sequence of events described above, that BMMF DNA itself will not be present in the precursor epithelial cells, nor in their malignant progeny. No evidence exists for the frequently quoted “Hit and Run” mechanism [27,28], since infected cells persist in the lamina propria, and oxygen radical production continues. The “Hits” in colon polyps continue from adjacent BMMF-infected cells (Figure 6) [29].

Does this mechanism provide predictable consequences for prevention and even therapy of such cancers?

Present recommendations for the prevention of colon cancer include avoiding consumption of red meat, avoidance of obesity, use of non-steroidal anti-inflammatory drugs, e.g., aspirin, cox-2 inhibitors and others [6]. Secondary prevention relies on colonoscopies, as well as the detection and removal of precursor lesions (colon polyps). Undeniably, this is a successful procedure, reducing the risk for subsequent colon cancer development. It requires, however, in several cases surgical intervention, in particular when removal of the polyp was only partially successful.

Our data point to possibilities for prevention even very early in life. Specific human milk sugars (*2′- and 3′-fucosyllactose* and *disialyl-lacto-N-tetraose*) occur in human, but not in cow milk [6]. These sugars bind to lectin receptors, thereby blocking the binding for several different infectious agents. Initial reports unraveled this effect for agents causing severe gastrointestinal and respiratory infections. Interestingly, where baby formulas had been supplemented with human milk sugars, a risk reduction was observed for acute childhood leukemias, Hodgkin’s disease and multiple sclerosis [6] in non-breast-fed or early-weaned babies. Their risk for developing these diseases then approximates to that of breast-fed babies [30]. A small number of mostly short-term studies reported analyses of the protective effects of these sugars when added to the diet of adults; the results remain inconclusive at present.

**Figure 7:**
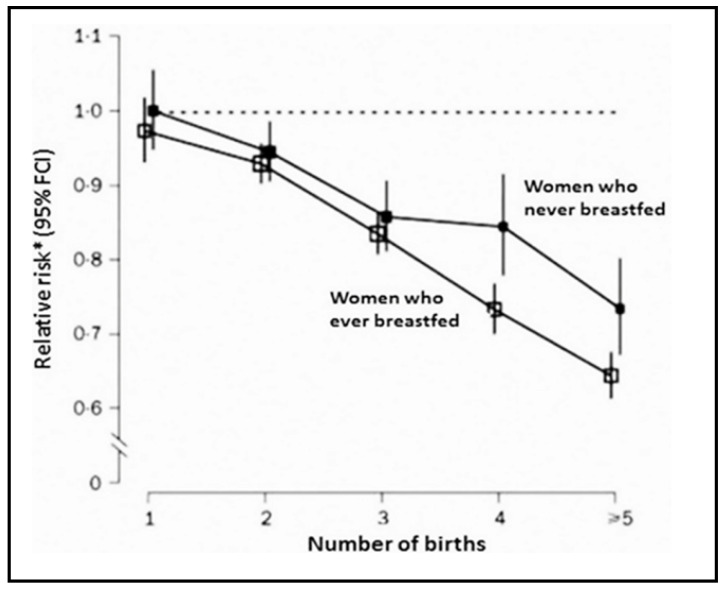
Reduced breast cancer risk after multiple deliveries and breast-feeding periods. Slightly less impressive reductions exist for colon and endometrial cancers (adapted with permission from [6]).

Indirect evidence for activity of the mentioned human sugars in adults originated, however, from follow-up studies of women with multiple pregnancies and breast-feeding periods (Figure 7). A significant reduction in breast cancer, but also in a few additional cancer types occurred in follow-up studies of this group [6]. Human milk sugars appeared in the blood and urine of these women, emerging during the second half of each pregnancy [31,32]. Other groups published similar observations and attempted to link this protective effect to specific hormonal interactions [33]. Yet, in our interpretation, this may provide a hint that prolonged exposure to human sugars in adults probably does not pose a high risk for the recipients and even seems to be protective. Although suggestive, the beneficial effects of the consumption of human milk sugar components at higher ages remain to be evaluated.

## 4. *N*-glycolylneuraminic Acid (*Neu5Gc)*

As suspected in a previous publication [16] Neu5Gc emerges as a strong candidate, as a terminal glycan of lectin receptors determining the susceptibility for various infections—in particular, those which are prevented by human milk sugars. We proposed blocking effects of the latter (probably due to their high affinity as terminal components of these receptors) for these infections as a mechanism of the observed protective effect of HMOs.

The nutritional transmission of Neu5Gc and the resulting immune response against this glycan [34,35] resulted in the speculation that reactive chronic immune reactions eventually induced mutational modifications in proliferating cells. This was proposed to explain malignant conversions following red meat consumption. The discovery of BMMF genomes and the expression of their Rep proteins in chronic inflammatory lesions of the colon and other tissues binding to the respective receptors added another dimension to these observations.

Among others, two questions emerge with relevance for the prevention of BMMF-linked cancers and other diseases:

Why are the cancers discussed here not at all, or extremely rarely, found in Eurasian dairy cattle, although BMMF infections are very common in their peripheral blood, their udders and in their milk?

The most likely answer seems to be an almost invariable immune tolerance resulting from the continuous conversion of Neu5Ac *(N*-acetylneuraminic acid) into Neu5Gc. Thus, this stresses the importance of inflammatory lesions in humans—their absence or reduction clearly leads to risk reductions for colon, breast, and prostate cancers. The subsequent chapter stresses this point.

It therefore also renders it difficult to include animal experiments for documenting BMMF carcinogenicity. Cattle, mice and most other mammals, as well as birds (except chicken) produce Neu5Gc endogenously. They are most likely immune tolerant. Thus, BMMF infection per se will not induce chronic inflammatory lesions.

**Figure 8:**
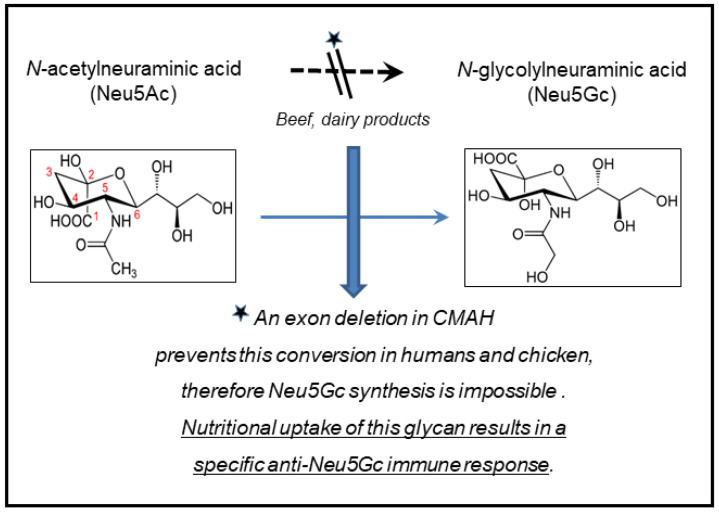
Cytidine monophosphate-N-acetylneuraminic acid hydrolase (CMAH).

The second question concerns an evolutionary aspect: is the loss of the Neu5Ac–Neu5Gc converting enzyme CMAH (Figure 8) an advantage for human evolution? To provide a clear-cut answer is probably difficult; it is unlikely that malignant tumors, commonly occurring late in life, will influence the selection of Neu5Gc-negative persons. Frequently fatal infections, however, in the first year or years of life, like noro- and rotavirus, gastrointestinal or respiratory tract infections, will almost certainly favor selection of CMAH-negative persons. Other types of prehistoric infections in the early period of human evolution may have added to this development.

Yet, it becomes increasingly clear (if we disregard possibilities of rare vertical trans-placenta transmissions) that we are commonly born without BMMF infections. Humans are even devoid of endogenous Neu5Gc, an essential component for binding BMMFs to cellular receptors. Thus, we receive both of these components through nutrition, mainly from dairy products and, in particular, Neu5Gc from dairy products and “red” meat. Both of these components offer possibilities for specific preventive and therapeutic interferences—they need to be further explored.

## 5. Non-Steroidal Antiphlogistica: Preventive and Immunosuppressive Events

**Figure 9:**
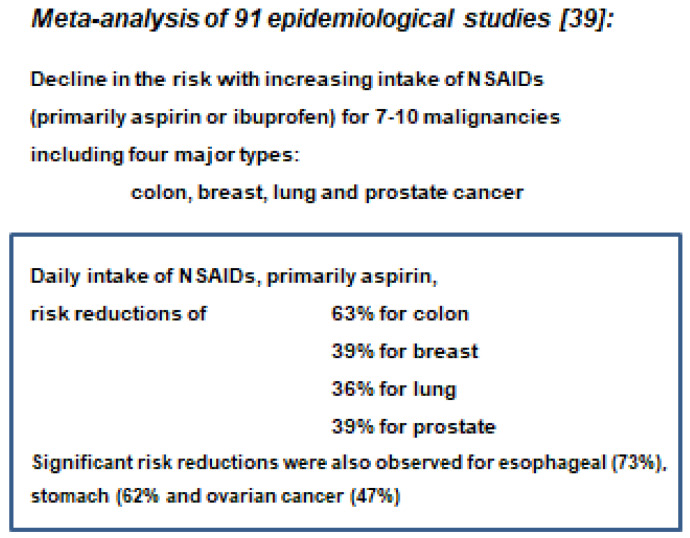
Significance of inflammatory events for specific human cancers, e.g., aspirin, ibuprofen and other Cox-2 inhibitors. Copied from [6] with permission.

Inflammatory events adjacent to malignant or premalignant tumors have repeatedly been implicated in playing a role in tumor initiation or promotion [5,36,37,38]. This has been underlined by the tumor-preventive functions of non-steroidal anti-inflammatory drugs (NSAIDs) (Figure 9), as analyzed in a meta-analysis by Harris et al. [39]. 

The anti-inflammatory function of these drugs results in blocking of the induction of mutagenic events induced by oxygen radicals, as described for the mode of action of indirect carcinogenesis [16,17]. Supporting evidence for this interpretation originates from the reduced risk for colon, breast and prostate cancers after prolonged immunosuppression in organ transplant patients or chronic infections with human immunodeficiency viruses [8]. Long-time intake of NSAIDs, in particular of higher doses of aspirin and related drugs, may cause bleeding risk and requires medical supervision.

## 6. Type II Diabetes Mellitus and BMMF

**Figure 10:**
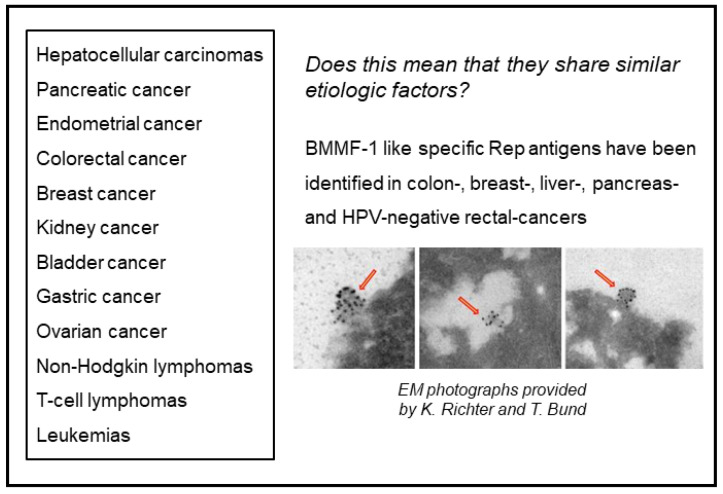
Cancers increased in type II Diabetes mellitus patients (summarized from [46]). The right lower part (red arrows) of the figure shows vesicular pleomorphic structures labelled with Rep antibody-coated gold particles, suspected to represent BMMF-structures.

As summarized previously, human milk oligosaccharides (HMO)–among other protective effects–also reduce the risk for early onset of diabetes mellitus [16]. A remarkably interesting observation resulted from treating type II diabetes mellitus patients with the glucagon-inhibitor *metformin*: intake of this drug reduced the formation of colon polyps. Based on the knowledge that the latter depends on inflammatory reactions caused by BMMF infections and CD68 macrophages, this stimulates the hypothesis that metformin may act as an inhibitor of BMMF synthesis [40,41,42,43,44,45]. This question at present remains unresolved. It is, however, an interesting observation that the incidence of several cancers is increased in type II Diabetes mellitus patients (Figure 10) [46,47,48].

We previously linked several of these cancers (among them colorectal and breast cancers) to indirect modes of carcinogenesis. We derived this conclusion from the detection and isolation of BMMF genomes from these tissues, or the positive staining for BMMF-1 Rep antigen (the latter also in Hepatitis B- and C-negative liver cancers, in prostate and HPV-negative rectal cancers—not shown in Figure 10).

Are they arising from mutational events triggered by oxygen or nitrogen radicals? Does this suggest common etiologic factors for these cancers? These cancers are clearly very different from those with increased incidence arising under immunosuppression [48].

**Figure 11:**
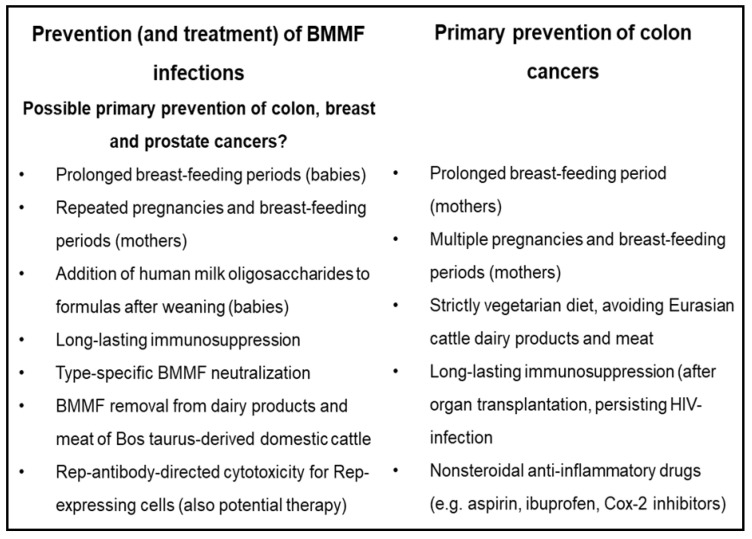
Several potential and established preventive interventions for colon cancer in comparison to possible preventions of BMMF infections.

Many of the outlined preventive or therapeutic interventions require intensive further investigation. Induction of long-lasting immunosuppression, mentioned as a preventive interference, is presently of no practical value for medical applications. Type-specific neutralization of BMMF, removal of BMMF from Eurasian-cattle products, as well as Rep-antibody-directed cytotoxicity of Rep-expressing cells as preventive measures of BMMF also require further experimentation (Figure 11).

Additional existing possibilities deserve an evaluation—as previously discussed. The functional expression of the enzyme CMAH (cytidine-monophosphate-N-acetylneuraminic acid hydrolase) [34], converting acetylneuraminic acid (Neu5Ac) into *N*-glycolylneuraminic acid (Neu5Gc) [35], was deleted in mice [49]. This deletion corresponds to the natural situation in humans where a mutation in CMAH does not permit Neu5Gc synthesis.

Neu5Gc seems to represent an essential component of receptors for BMMF binding [6,16]. Theoretically, similar CMAH mutations induced by gene manipulation into breeds of Eurasian dairy cattle should result in Neu5Gc-negative cattle. The products from these animals should not pose a risk for BMMF infection of humans in the absence of appropriate receptors.

Rep antibody-directed cytotoxicity may have some therapeutic value, which needs further exploration. A possibly even more interesting alternative would be their use in destroying Rep-positive persistently BMMF-infected carrier cells by this procedure, even at an early stage of life. Prevention of the carrier state could represent a kind of “early stage secondary prevention” in already infected persons.

## 7. Basal Cell Carcinomas in Pox Vaccination Scars

**Figure 12:**
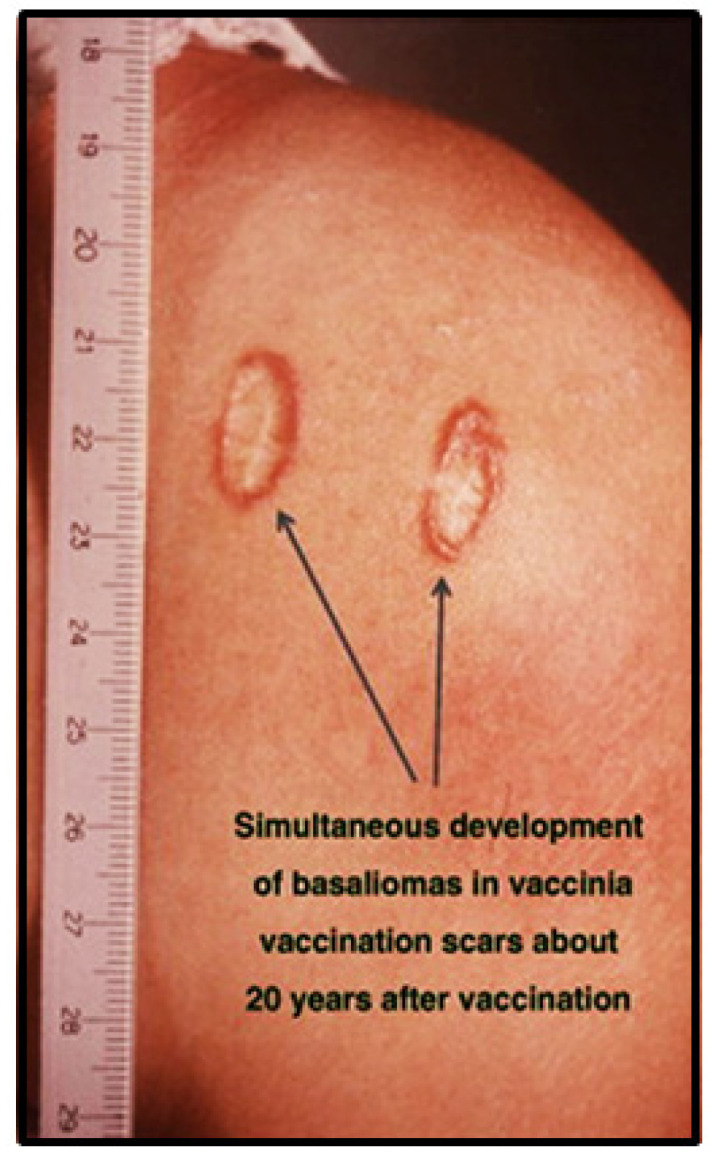
Basal cell carcinoma emerged in a pox vaccination scar. Copied [7] (with permission).

As previously mentioned, basal cell carcinomas (BCCs) can develop in pox vaccination scars (Figure 12) [7]. They may deserve special interest if a possible relationship to BMMF infections exists. Skin cancers occur in excess of one in three newly diagnosed cancers. BCCs represent the vast majority of skin cancers [7]. Prolonged solar exposure is a major risk factor for the development of actinic keratoses—precursor lesions for this cancer [50]. Occasional reports claim a role for several types of cutaneous papillomaviruses in addition to solar exposure in the pathogenesis of these malignancies. This has then frequently been referred to as a “hit and run” mechanism. This postulate tried to explain inconsistencies—in particular, the absence of persisting viral DNA in these cancer cells [1,27,28]. A few observations on BCCs reported over past decades may provide clues deserving a fresh look; BCCs repeatedly emerged in pox vaccination scars, occasionally as multiple foci, years after inoculation with the vaccine [7].

Studies performed by our group during the past decade provide the basis for a different speculation: we demonstrated an almost regular presence of CD68-positive TAMs in foci of BMMF1 Rep-positive peri-tumorous cells [16]. During previous decades, poxvirus vaccines were prepared from inoculation of vaccinia virus preparations into the scratched skin of calves. The initial formation of pox-like pustules subsequently converted to crusts. The copious vaccinia virus crudely purified from these harvested crusts represented the basis for human vaccination.

Presently, we know that the majority of Eurasian cattle are carriers of BMMF infections in various tissues, including peripheral blood [9,17]. BMMF transmission/transfusion into the scratches on the calfskin is highly probable. The intensive local infection occurring after vaccination and the subsequent induction of slow chronic inflammation many years after the inoculation of the vaccine could result in BCCs in the vaccination scars. Other environmental mutagens may synergize with this process [7].

## 8. Acute Myeloid Leukemia

**Figure 13:**
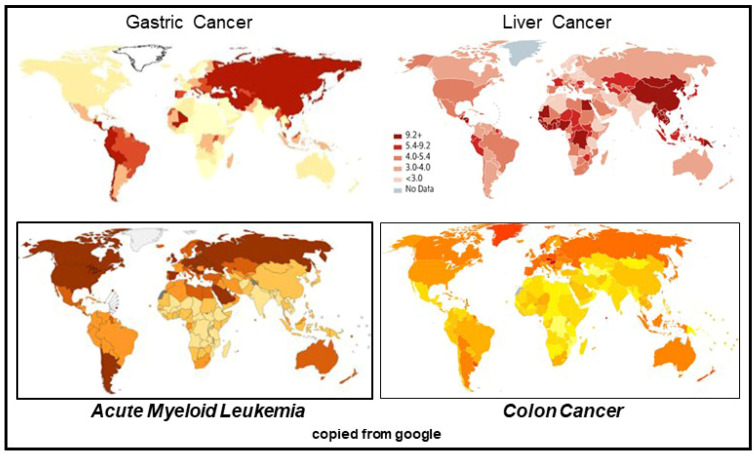
Global epidemiological incidence of AML and colon cancer in comparison to gastric and liver cancers.

In addition to the global epidemiological incidence pattern of BMMF infection-linked cancers discussed up to now, one other neoplastic disease deserves further investigation: acute myeloid leukemia (AML). The global incidence of this malignancy is somewhat similar to the global pattern of colon cancer incidence (Figure 13). The figure compares the global pattern of those two cancers with the very different epidemiology of gastric and liver cancers.

RNA-seq data provided evidence for BMMF1 transcription. These sequences differ substantially from the BMMF1 HSB1 in colon cancers [51]. These data seem to be of specific interest for several reasons:

1. To our knowledge, this is the first direct link between a human leukemia and a specific BMMF infection;
2. Since the respective BMMF types have been identified in dairy products, it is likely that the human infection was acquired by consumption of infected nutritional components;
3. AML, as well as acute lymphatic leukemia (ALL), has very often been reported to occur even in the prenatal phase. A further confirmation of their infectious origin implies prenatal trans-placenta transmission during pregnancy, as proposed previously [52,53].

Future transcription analyses of acute lymphatic leukemias searching for BMMF-like infections will be of substantial interest.

## 9. Conclusions

Except for infection-linked human cancers (EBV, HPV, HBV, HCV, others) our knowledge of agents causally involved in the majority of human cancers is still very limited. This applies for chronic neurological diseases, arteriosclerosis, autoimmune and metabolic diseases as well. A fresh look into indirect mechanisms, as defined now for a few common cancers, may provide surprising results, with far reaching implications for the prevention and therapy of these diseases.
